# Supplementary material for: Simultaneous Qualitative and Quantitative Evaluation of the Coptidis Rhizoma and Euodiae Fructus Herbal Pair by Using UHPLC-ESI-QTOF-MS and UHPLC-DAD
Source: Molecules. 2020 Oct 18;25(20):4782. doi: 10.3390/molecules25204782 (PMC7587604; doi:10.3390/molecules25204782)
Supplement: Supplementary file 1 [file molecules-25-04782-s001.pdf]

Supplementary Materials: Simultaneous qualitative and quantitative evaluation of Coptidis Rhizoma and Euodiae Fructus herbal pair by using UHPLC-ESI-QTOF-MS and UHPLC-DAD

Yan-Ying Li, Min-Qun Guo, Xue-Mei Li, Xiu-Wei Yang

Table S1. Characterization of compounds in the extract of CR-EF herbal pair determined by UHPLC-ESI-QTOF-MS/MS

No.	t_R (min)	Formula	Identification	Ion mode	Mass (m/z) (Δ ppm)	Fragment ions (m/z)	Type	Resources
**1**	2.800	$C_{13}H_{16}O_9$	Gentisic acid 5-*O*-β-D-glucoside	$[M–H]^-$	315.0721 (0.18)	315.0726 $[M–H]^-$, 152.0107 $[M–H–Glc]^-$, 108.0212 $[M–H–Glc–HCOO]^-$	PH	CR
**2**	7.038	$C_{10}H_{12}O_5$	Danshensu methyl ester	$[M–H]^-$	211.0612 (–0.01)	151.8873 $[M–H–CH_3COOH]^-$, 123.0458 $[M–H–CH_3COOH–CO]^-$	PH	CR
**3**	11.684	$C_{20}H_{24}NO_4^+$	Magnoflorine	$[M]^+$	342.1703 (–0.92)	342.1711 $[M]^+$, 297.1130 $[M–(CH_3)_2NH]^+$, 282.0894 $[M–(CH_3)_2NH–CH_3]^+$, 265.0865 $[M–(CH_3)_2NH–CH_3OH]^+$	AD	CR
**4**	12.511	$C_{17}H_{20}O_9$	3-*O*-Caffeoylquinic acid methyl ester	$[M–H]^-$	367.1034 (0.15)	367.1044 $[M–H]^-$, 179.0331 [Caffeic acid–H]$^-$, 161.0248 [Caffeic acid–H–H_2O]$^-$, 133.0299 [Caffeic acid–H–HCOOH]$^-$	PH	EF

**5**	13.762	$C_{16}H_{20}O_{10}$	Caffeoyl-6-*O*-D-gluconic acid methylester	$[M–H]^–$	371.0986 (–0.62)	371.1014 $[M–H]^–$, 339.0732 $[M–H–CH_3OH]^–$, 179.0358 [Caffeic acid–H]$^–$, 161.0253 [Caffeic acid–H–H_2O]$^–$, 135.0473 [Caffeic acid–H–CO_2]$^–$	PH	EF
**6**	18.778	$C_{15}H_{16}O_9$	Caffeoyl-6-*O*-D-glucono-γ-lactone	$[M–H]^–$	339.0718 (1.05)	339.0713 $[M–H]^–$, 179.0343 [Caffeic acid–H]$^–$, 161.0256 [Caffeic acid–H–H_2O]$^–$, 135.0442 [Caffeic acid–H–CO_2]$^–$	PH	EF
**7**	19.113	$C_{26}H_{34}O_{11}$	(+)-lariciresinol glucoside	$[M–H]^–$	521.2030 (–0.32)	521.2042 $[M–H]^–$, 359.1508 [M–H–Glc]$^–$, 344.1276 [M–H–Glc–CH_3]$^–$	PH	CR
**8**	19.743	$C_{16}H_{20}O_9$	methyl 4-*O*-β-D-glucosyl caffeate	$[M–H]^–$	355.1029 (1.57)	193. 0505 [M–H–Glc]$^–$, 161.0260 [Caffeate–H_2O]$^–$, 134.0349 [M–H–Glc–CH_3COO]$^–$	PH	EF
**9**	20.402	$C_{17}H_{20}O_9$	4-*O*-Caffeoylquinic acid methyl ester	$[M–H]^–$	367.1032 (0.7)	367.1028 $[M–H]^–$, 161.0244 $[M–H–C_8H_{12}O_5–H_2O]^–$, 135.0449 $[M–H– C_8H_{12}O_5–CO_2]^–$,	PH	EF
**10**	21.612	$C_{18}H_{22}O_9$	3-*O*-feruloylquinic acid methyl ester	$[M+H]^+$	383.1340 (–0.89)	383.1368 $[M+H]^+$, 177.0538 [Feruloyl]$^+$, 145.0274 [Feruloyl–CH_3OH]$^+$, 117.0333 [Feruloyl–CH_3OH–CO]$^+$	PH	CR
**11**	22.216	$C_{18}H_{16}NO_4^+$	9,10-dihydroxycolumbamine	$[M]^+$	310.1075 (–0.37)	310.1072 $[M]^+$, 295.0843 $[M–CH_3]^+$, 267.0884 $[M–CH_3–CO]^+$	AD	CR
**12**	22.461	$C_{20}H_{18}NO_5^+$	Berberastine	$[M]^+$	352.1179 (0.14)	352.1171 $[M]^+$, 334.1067 $[M–H_2O]^–$,	AD	CR

						320.0913 [M–H_2O–CH_2]$^+$,<br>292.0938 [M–H_2O–CH_2–CO]$^+$		
**13**	23.946	$C_{20}H_{19}NO_5$	Sinotumine H	[M+H]$^+$	354.1334<br>(0.56)	354.1336 [M+H]$^+$,<br>339.1096 [M+H–CH_3]$^+$,<br>338.1013 [M+H–CH_4]$^+$,<br>324.0858 [M+H–CH_4–CH_2]$^+$,<br>310.1070 [M+H–CH_4 CO]$^+$,<br>296.0881 [M+H–CH_4–CH_2–CO]$^+$,<br>278.0747 [M+H–CH_4–CH_2–CO–CH_4]$^+$	AD	CR
**14**	25.144	$C_{17}H_{20}O_9$	5-*O*-Caffeoyl quinic acid methyl ester	[M–H]$^-$	367.1036<br>(–0.39)	367.1044 [M–H]$^-$,<br>179.0350 [Caffeic acid–H]$^-$,<br>161.0261 [Caffeic acid–H–H_2O]$^-$,<br>135.0442 [Caffeic acid–H–CO_2]$^-$	PH	EF
**15**	26.404	$C_{20}H_{17}NO_5$	8-Oxoepiberberine	[M+H]$^+$	352.1184<br>(–1.28)	352.1186 [M+H]$^+$,<br>336.0871 [M+H–CH_4]$^+$,<br>322.0718 [M+H–CH_4–CH_2]$^+$,<br>308.0924 [M+H–CH_4–CO]$^+$,<br>294.0758 [M+H–CH_4–CH_2–CO]$^+$	AD	CR
**16**	26.937	$C_{19}H_{16}NO_4^+$	Berberrubine	[M]$^+$	322.1074<br>(–0.05)	322.1074 [M]$^+$,<br>307.0841 [M–CH_3]$^+$,<br>294.0772 [M–CO]$^+$,<br>279.0885 [M–CH_3–CO]$^+$	AD	CR
**17**	27.652	$C_{19}H_{18}NO_4^+$	2-Hydroxyjatrorrhizine	[M]$^+$	324.1229<br>(0.41)	324.1231 [M]$^+$,<br>308.0914 [M–CH_4]$^+$,<br>294.0761 [M–CH_4–CH_2]$^+$,<br>280.0963 [M–CH_4–CO]$^+$,<br>266.0805 [M– CH_4–CH_2–CO]$^+$	AD	CR
**18**	28.933	$C_{19}H_{16}N_3O^+$	Dehydroevodiamine	[M]$^+$	302.1284<br>(1.29)	302.1290 [M]$^+$,<br>287.1046 [M–CH_3]$^-$,	AD	EF

						286.0978 $[M–CH_4]^+$, 272.0819 $[M–CH_3NH]^+$, 258.1024 $[M–C_2H_4O]^+$		
**19**	29.421	$C_{10}H_{10}O_4$	Methyl caffeate	$[M–H]^–$	193.0510 (–1.9)	193.0500 $[M–H]^–$, 160.8414 $[M–H–OCH_3–H_2]^–$, 134.0362 $[M–H–OCH_3–CO]^–$, 133.0283 $[M–H–CH_3COOH]^–$	PH	EF
**20**	30.302	$C_{18}H_{14}NO_4^+$	Demethylene coptisine	$[M]^+$	308.0916 (0.44)	308.0918 $[M]^+$, 293.0678 $[M–CH_3]^+$, 280.0951 $[M–CO]^+$, 265.0733 $[M–CH_3–CO]^+$	AD	CR
**21**	30.776	$C_{18}H_{22}O_9$	4-*O*-feruloylquinic acid methyl ester	$[M+H]^+$	383.1336 (0.15)	177.0539 [feruloyl]$^+$, 145.0278 [feruloyl–CH_3OH]$^+$, 117.0332 [feruloyl–CH_3OH–CO]$^+$	PH	CR
**22**	32.094	$C_{19}H_{14}NO_4^+$	Coptisine	$[M]^+$	320.0920 (–0.83)	320.0932 $[M]^+$, 292.0976 $[M–CO]^+$, 277.0746 $[M–CO–CH_3]^+$, 262.0873 $[M–CO–2CH_3]^+$	AD	CR
**23**	33.975	$C_{19}H_{18}NO_4^+$	2-Hydroxyjatrorrhizine isomer	$[M]^+$	324.1237 (–0.82)	324.1230 $[M]^+$, 308.0917 $[M–CH_4]^+$, 280.0960 $[M–CH_4–CO]^+$	AD	CR
**24**	35.472	$C_{19}H_{16}NO_4^+$	Groenlandicine isomer	$[M]^+$	322.1080 (–1.91)	322.1084 $[M]^+$, 307.0848 $[M–CH_3]^+$, 279.0890 $[M–CH_3–CO]^+$	AD	CR
**25**	38.425	$C_{20}H_{18}NO_4^+$	Epiberberine	$[M]^+$	336.1229 (0.4)	336.1238 $[M]^+$, 320.0925 $[M–CH_4]^+$, 292.0974 $[M–CH_4–CO]^+$	AD	CR
**26**	40.052	$C_{20}H_{20}NO_4^+$	Columbamine	$[M]^+$	338.1387 (–0.05)	338.1392 $[M]^+$, 322.1086 $[M–CH_4]^+$, 308.0929 $[M–CH_4–CH_2]^+$, 294.1135 $[M–CH_4–CO]^+$,	AD	CR

						280.0970 $[M–CH_4–CH_2–CO]^+$		
**27**	41.162	$C_{20}H_{20}NO_4^+$	Jatrorrhizine	$[M]^+$	338.1390 (–0.93)	338.1395 $[M]^+$, 322.1083 $[M–CH_4]^+$, 308.0924 $[M–CH_4–CH_2]^+$, 294.1130 $[M–CH_4–CO]^+$, 280.0964 $[M–CH_4–CO–CH_2]^+$	AD	CR
**28**	42.468	$C_{18}H_{22}O_9$	5-*O*-feruloylquinic acid methyl ester	$[M+H]^+$	383.1344 (–1.93)	383.1338 $[M+H]^+$, 177.0550 $[Feruloyl]^+$, 145.0286 $[Feruloyl–CH_3OH]^+$, 117.0335 $[Feruloyl–CH_3OH–CO]^+$	PH	CR
**29**	44.760	$C_{19}H_{16}NO_4^+$	Groenlandicine	$[M]^+$	322.1086 (–0.67)	322.1082 $[M]^+$, 307.0848 $[M–CH_3]^+$, 279.0893 $[M–CH_3–CO]^+$	AD	CR
**30**	49.402	$C_{20}H_{18}NO_4^+$	Berberine	$[M]^+$	336.1253 (–0.97)	336.1241 $[M]^+$, 320.0924 $[M–CH_4]^+$, 306.0773 $[M–CH_4–CH_2]^+$, 292.0974 $[M–CH_4–CO]^+$, 278.0818 $[M–CH_4–CH_2–CO]^+$	AD	CR
**31**	55.350	$C_{21}H_{22}NO_4^+$	Palmatine	$[M]^+$	352.1546 (–0.75)	352.1550 $[M]^+$, 336.1235 $[M–CH_4]^+$, 322.1080 $[M–CH_4–CH_2]^+$, 308.1284 $[M–CH_4–CO]^+$, 294.1125 $[M–CH_4–CH_2–CO]^+$, 278.0814 $[M–2CH_4–CH_2–CO]^+$, 264.1018 $[M–2CH_4–2CH_2–CO]^+$	AD	CR
**32**	58.087	$C_{21}H_{20}NO_4^+$	13-Methylberberine	$[M]^+$	350.1389 (–0.62)	350.1385 $[M]^+$, 334.1068 $[M–CH_4]^+$, 320.1014 $[M–CH_4–CH_2]^+$, 306.1124 $[M–CH_4–CO]^+$, 292.0965 $[M–CH_4–CH_2–CO]^+$	AD	CR
**33**	58.248	$C_{28}H_{32}O_{15}$	Diosmin	$[M+H]^+$	609.1818	609.1808 $[M+H]^+$,	PH	EF

					(–0.66)	463.1235 $[M+H–Rha]^+$, 301.0706 $[M+H–Rha–Glc]^+$		
**34**	62.879	$C_{31}H_{27}NO_8$	Coptichine	$[M+H]^+$	542.1812 (–0.47)	334.1086 $[C_{20}H_{16}NO_4]^+$, 319.0843 $[C_{20}H_{16}NO_4–CH_3]^+$, 304.0617 $[C_{20}H_{16}NO_4–2CH_3]^+$, 290.0822 $[C_{20}H_{16}NO_4–2CH_3–CH_2]^+$	AD	CR
**35**	63.166	$C_{31}H_{27}NO_8$	Coptichine isomer	$[M+H]^+$	542.1816 (–1.21)	334.1067 $[C_{20}H_{16}NO_4]^+$, 319.0829 $[C_{20}H_{16}NO_4–CH_3]^+$, 304.0596 $[C_{20}H_{16}NO_4–2CH_3]^+$, 290.0806 $[C_{20}H_{16}NO_4–2CH_3–CH_2]^+$	AD	CR
**36**	66.032	$C_{26}H_{30}O_9$	Rutaevine	$[M+H]^+$	487.1964 (–0.29)	487.1946 $[M+H]^+$, 469.1846 $[M+H–H_2O]^+$, 443.2052 $[M+H–CO_2]^+$, 441.1879 $[M+H–H_2O–CO]^+$, 161.0578 $[C_9H_5O_3]^+$, 95.0127 $[C_5H_3O_2]^+$	LD	EF
**37**	67.026	$C_{26}H_{30}O_9$	12α-Hydroxylimonin	$[M+H]^+$	487.1962 (0.12)	487.1966 $[M+H]^+$, 469.1859 $[M+H–H_2O]^+$, 443.2064 $[M+H–CO_2]^+$, 441.1914 $[M+H–H_2O–CO]^+$, 161.0594 $[C_9H_5O_3]^+$, 95.0133 $[C_5H_3O_2]^+$	LD	EF
**38**	67.974	$C_{26}H_{30}O_8$	Limonin	$[M+H]^+$	471.2015 (–0.33)	471.2016 $[M+H]^+$, 425.1955 $[M+H–HCOOH]^+$, 161.0590 $[C_9H_5O_3]^+$, 95.0127 $[C_5H_3O_2]^+$	LD	EF
**39**	68.132	$C_{21}H_{19}NO_6$	8-Methoxyberberine phenolbetaine	$[M+H]^+$	382.1285 (0.04)	382.1264 $[M+H]^+$, 367.1038 $[M+H–CH_3]^+$, 350.1003 $[M+H–CH_3–OH]^+$, 335.0769 $[M+H–2CH_3–OH]^+$,	AD	CR

						320.0541 [M+H–3CH$_3$–OH]$^+$		
**40**	68.702	$C_{26}H_{28}O_9$	Evodol	[M+H]$^+$	485.1804 (0.43)	485.1795 [M+H]$^+$, 441.1891 [M+H–CO$_2$]$^+$, 423.1776 [M+H–CO$_2$–H$_2$O]$^+$, 381.1695 [M+H–CO$_2$–AcOH]$^+$, 353.1743 [M+H–CO$_2$–AcOH–CO]$^+$, 161.0594 [C$_9$H$_5$O$_3$]$^+$, 95.0125 [C$_5$H$_3$O$_2$]$^+$	LD	EF
**41**	68.815	$C_{18}H_{19}N_3O$	*N*^{b}-demethylevodiamide	[M+H]$^+$	294.1602 (–0.38)	294.1596 [M+H]$^+$, 134.0591 [C$_8$H$_8$NO]$^+$, 116.0485 [C$_8$H$_6$N]$^+$, 106.0642 [C$_7$H$_8$N]$^+$, 91.0535 [C$_6$H$_5$N]$^+$	AD	EF
**42**	68.860	$C_{19}H_{21}N_3O$	Evodiamide	[M+H]$^+$	308.1761 (–1.17)	308.1756 [M+H]$^+$, 134.0603 [C$_8$H$_8$NO]$^+$, 116.0500 [C$_8$H$_6$N]$^+$, 106.0655 [C$_7$H$_8$N]$^+$, 91.0549 [C$_6$H$_5$N]$^+$	AD	EF
**43**	69.014	$C_{26}H_{30}O_{10}$	Glaucin A	[M+H]$^+$	503.2266 (>5)	503.2273 [M+H]$^+$, 457.2216 [M+H–HCOOH]$^+$, 161.0594 [C$_9$H$_5$O$_3$]$^+$, 95.0133 [C$_5$H$_3$O$_2$]$^+$	LD	EF
**44**	69.201	$C_{20}H_{17}NO_5$	8-Oxoberberine	[M+H]$^+$	352.1184 (–1.28)	352.1186 [M+H]$^+$, 337.0951 [M+H–CH$_3$]$^+$, 322.0713 [M+H–CH$_4$–CH$_2$]$^+$, 308.0917 [M+H–C$_2$H$_4$O]$^+$, 294.0763 [M+H–CH$_4$–CH$_2$–CO]$^+$	AD	CR
**45**	69.376	$C_{27}H_{32}O_{10}$	Evorubodinin	[M–H]$^-$	515.1918 (0.91)	515.1934 [M–H]$^-$, 427.1409 [M–H–2CO$_2$]$^-$, 401.0887 [M–H–C$_5$H$_6$O$_3$]$^-$, 357.0601 [M–H–C$_5$H$_6$O$_3$–CO$_2$]$^-$,	LD	EF

						313.0738 $[M–H–C_5H_6O_3–2CO_2]^–$, 269.0830 $[M–H–C_5H_6O_3–2CO_2–CH_4–CO]^–$		
**46**	69.455	$C_{19}H_{17}N_3O$	Evodiamine	$[M+H]^+$	304.1449 (–1.52)	304.1427 $[M+H]^+$, 171.0902 $[M+H–C_8H_7NO]^+$, 134.0590 $[M+H–C_{11}H_{10}N_2]^+$	AD	EF
**47**	69.696	$C_{28}H_{32}O_{10}$	6-Acetoxy-5-epilimonin	$[M+H]^+$	529.2065 (0.42)	529.2056 $[M+H]^+$, 485.2151 $[M+H–CO_2]^+$, 451.1742 $[M+H–AcOH–H_2O]^+$, 425.1947 $[M+H–CO_2–AcOH]^+$, 393.1317 $[M+H–CO_2–AcOH–2CH_4]^+$, 367.1527 $[M+H–CO_2–AcOH–2CH_3–CO]^+$, 161.0585 $[C_9H_5O_3]^+$, 95.0129 $[C_5H_3O_2]^+$	LD	EF
**48**	69.859	$C_{18}H_{13}N_3O$	Rutaecarpine	$[M+H]^+$	288.112 (0.83)	288.1143 $[M+H]^+$, 273.0905 $[M+H–CH_3]^+$, 244.0887 $[M+H–CH_4–CO]^+$, 169.0762 $[C_{11}H_8N_2+H]^+$	AD	EF
**49**	70.067	$C_{19}H_{15}N_3O_2$	Hortiacine	$[M+H]^+$	318.1239 (–0.62)	318.1243 $[M+H]^+$, 286.0981 $[M+H–CH_3OH]^+$, 258.1023 $[[M+H–CH_3OH–CO]^+$, 167.0609 $[C_{11}H_6N_2+H]^+$	AD	EF
**50**	70.586	$C_{19}H_{19}N_3O$	Goshuyuamide I	$[M+H]^+$	306.1599 (0.62)	306.1584 $[M+H]^+$, 144.0803 $[C_{10}H_{10}N]^+$, 134.0595 $[C_8H_7NO+H]^+$, 116.0491 $[C_8H_5N+H]^+$, 106.0647 $[C_7H_7N+H]^+$	AD	EF
**51**	71.381	$C_{19}H_{25}NO$	1-Methyl-2-[(Z)-4-nonenyl]-4(1*H*)-quinolone	$[M+H]^+$	284.2007 (0.67)	284.2005 $[M+H]^+$, 200.1077 $[C_{10}H_9NO+C_3H_5]^+$,	AD	EF

						186.0906 $[C_{10}H_9NO+C_2H_3]^+$, 173.0828 $[C_{10}H_9NO+CH_2]^+$, 159.0671 $[C_{10}H_9NO]^+$		
**52**	71.705	$C_{23}H_{35}NO_2$	1-Methyl-2-[12-hydroxy-tridecyl]-4(1H)-quinolone	$[M+H]^+$	358.2739 (0.43)	358.2728 $[M+H]^+$, 340.2624 $[M+H–H_2O]^+$, 186.0915 $[C_{10}H_9NO+C_2H_3]^+$, 173.0823 $[C_{10}H_9NO+CH_2]^+$	AD	EF
**53**	72.837	$C_{19}H_{27}NO$	1-Methyl-2-nonyl-4(1*H*)-quinolone	$[M+H]^+$	286.2167 (–0.56)	286.2165 $[M+H]^+$, 186.0911 $[C_{10}H_9NO+C_2H_3]^+$, 173.0835 $[C_{10}H_9NO+CH_2]^+$, 159.0669 $[C_{10}H_9NO]^+$	AD	EF
**54**	73.560	$C_{21}H_{29}NO$	1-Methyl-2-[(*Z*)-6-undecenyl]-4(1*H*)-quinolone	$[M+H]^+$	312.2323 (–0.35)	312.2313 $[M+H]^+$, 228.1375 $[M+H–C_6H_{12}]^+$, 200.1061 $[M+H–C_8H_{16}]^+$, 186.0909 $[C_{10}H_9NO+C_2H_3]^+$, 173.0829 $[C_{10}H_9NO+CH_2]^+$, 159.0673 $[C_{10}H_9NO]^+$	AD	EF
**55**	74.326	$C_{23}H_{31}NO$	1-Methyl-2-[(4*Z*,7*Z*)- tridecadienyl]-4(1*H*)-quinolone	$[M+H]^+$	338.2478 (0.12)	338.2481 $[M+H]^+$, 212.1065 $[M+H–C_9H_{18}]^+$, 186.0909 $[C_{10}H_9NO+C_2H_3]^+$, 173.0831 $[C_{10}H_9NO+CH_2]^+$, 159.0675 $[C_{10}H_9NO]^+$	AD	EF
**56**	74.675	$C_{21}H_{29}NO$	1-Methyl-2-[(*E*)-1-undecenyl]-4(1*H*)-quinolone	$[M+H]^+$	312.2329 (–2.27)	312.2319 $[M+H]^+$, 198.0917 $[M+H–C_8H_{18}]^+$, 184.0760 $[C_{10}H_9NO+C_2H_3]^+$, 173.0831 $[C_{10}H_9NO+CH_2]^+$, 159.0679 $[C_{10}H_9NO]^+$	AD	EF
**57**	74.792	$C_{21}H_{31}NO$	1-Methyl-2-undecyl-4(1*H*)-quinolone	$[M+H]^+$	314.2482 (–1.14)	314.2474 $[M+H]^+$, 200.1060 $[C_{10}H_9NO+C_3H_5]^+$, 186.0901 $[C_{10}H_9NO+C_2H_3]^+$, 173.0826 $[C_{10}H_9NO+CH_2]^+$, 159.0661 $[C_{10}H_9NO]^+$	AD	EF

**58**	74.954	$C_{25}H_{33}NO$	1-Methyl-2-[(6*Z*, 9*Z*, 12*E*)-pentadeca triene]-4(1*H*)-quinolone	$[M+H]^+$	364.2631 (1.07)	364.2640 $[M+H]^+$, 308.2008 $[M+H–C_4H_8]^+$, 268.1688 $[M+H–C_7H_{12}]^+$, 228.1375 $[M+H–C_{10}H_{16}]^+$, 200.1064 $[C_{10}H_9NO+C_3H_5]^+$, 186.0909 $[C_{10}H_9NO+C_2H_3]^+$, 173.0830 $[C_{10}H_9NO+CH_2]^+$, 159.0671 $[C_{10}H_9NO]^+$	AD	EF
**59**	75.274	$C_{23}H_{33}NO$	Evocarpine	$[M+H]^+$	340.2638 (–0.91)	340.2652 $[M+H]^+$, 256.1704 $[M+H–C_6H_{12}]^+$, 242.1547 $[M+H–C_7H_{14}]^+$, 228.1389 $[M+H–C_8H_{16}]^+$, 186.0923 $[C_{10}H_9NO+C_2H_3]^+$, 173.0844 $[C_{10}H_9NO+CH_2]^+$, 159.0684 $[C_{10}H_9NO]^+$	AD	EF
**60**	75.649	$C_{25}H_{35}NO$	1-Methyl-2-[(6*Z*,9*Z*)-pentadecadienyl]-4(1*H*)-quinolone	$[M+H]^+$	366.2796 (–1.25)	366.2791 $[M+H]^+$, 268.1691 $[M+H–C_7H_{14}]^+$, 228.1378 $[M+H–C_{10}H_{18}]^+$, 186.0910 $[C_{10}H_9NO+C_2H_3]^+$, 173.0833 $[C_{10}H_9NO+CH_2]^+$, 159.0670 $[C_{10}H_9NO]^+$	AD	EF
**61**	75.969	$C_{24}H_{35}NO$	1-Methyl-2-[13-tetradecenyl]-4-(1*H*)-quinolone	$[M+H]^+$	354.2794 (–0.73)	354.2801 $[M+H]^+$, 200.1071 $[C_{10}H_9NO+C_3H_5]^+$, 186.0911 $[C_{10}H_9NO+C_2H_3]^+$, 173.0836 $[C_{10}H_9NO+CH_2]^+$, 160.0774 $[C_{10}H_9NO+H]^+$, 159.0665 $[C_{10}H_9NO]^+$	AD	EF
**62**	76.439	$C_{23}H_{35}NO$	Dihydroevocarpine	$[M+H]^+$	342.2794 (–0.76)	342.2791 $[M+H]^+$, 200.1066 $[C_{10}H_9NO+C_3H_5]^+$, 186.0913 $[C_{10}H_9NO+C_2H_3]^+$, 173.0833 $[C_{10}H_9NO+CH_2]^+$, 159.0668 $[C_{10}H_9NO]^+$	AD	EF

**63**	76.684	$C_{25}H_{37}NO$	1-Methyl-2-[(*Z*)-10-pentadecenyl]-4(1*H*)-quinolone	$[M+H]^+$	368.2973 (–0.84)	368.2953 $[M+H]^+$,<br>228.1383 $[M+H–C_{10}H_{20}]^+$,<br>200.1063 $[C_{10}H_9NO+C_3H_5]^+$,<br>186.0911 $[C_{10}H_9NO+C_2H_3]^+$,<br>173.0831 $[C_{10}H_9NO+CH_2]^+$,<br>159.0675 $[C_{10}H_9NO]^+$	AD	EF
**64**	77.196	$C_{24}H_{37}NO$	1-Methyl-2-tetradecy-4-(1*H*)-quinolone	$[M+H]^+$	356.2952 (–1.15)	356.2953 $[M+H]^+$,<br>228.1366 $[M+H–C_9H_{20}]^+$,<br>200.1057 $[C_{10}H_9NO+C_3H_5]^+$,<br>186.0909 $[C_{10}H_9NO+C_2H_3]^+$,<br>173.0835 $[C_{10}H_9NO+CH_2]^+$,<br>159.0656 $[C_{10}H_9NO]^+$	AD	EF
**65**	78.061	$C_{25}H_{39}NO$	1-Methyl-2-pentadecyl-4(1*H*)-quinolone	$[M+H]^+$	370.3107 (–0.7)	370.3110 $[M+H]^+$,<br>200.1069 $[C_{10}H_9NO+C_3H_5]^+$,<br>186.0918 $[C_{10}H_9NO+C_2H_3]^+$,<br>173.0838 $[C_{10}H_9NO+CH_2]^+$,<br>159.0676 $[C_{10}H_9NO]^+$	AD	EF

CR: Coptidis Rhizoma; EF: Euodiae Fructus; AD: alkaloids; LD: limonoids; PH: phenolics.

Table S2. Contents of the nine analytes in 19 samples ($mg \cdot g^{-1}$)

Sample No.	Coptisine	Epiberberine	Columbamine	Jatrorrhizine	Berberine	Palmatine	Dehydroevodiamine	Evodiamine	Rutaecarpine
S2	13.15	8.53	4.25	3.23	36.83	7.89	1.63	0.21	0.16
S3	11.10	6.78	4.00	2.82	37.09	8.13	1.50	0.15	0.13
S4	15.36	8.66	4.47	2.90	36.23	8.60	1.67	1.73	0.87
S5	16.06	7.40	7.18	4.66	42.99	12.14	2.02	0.22	0.15
S6	17.67	8.36	4.35	2.71	39.26	9.39	2.00	0.19	0.15
S7	15.22	8.50	6.23	4.02	40.63	10.67	1.56	1.49	0.82
S8	14.77	10.68	6.42	3.80	37.05	9.96	2.12	0.25	0.17
S9	15.73	9.22	5.10	3.24	42.71	9.05	1.96	1.69	0.94
S10	16.55	10.51	4.59	3.45	33.41	8.51	1.74	0.45	0.27
S11	12.78	8.11	9.16	4.69	41.99	13.30	1.73	3.71	1.74

S12	18.52	11.85	3.91	3.41	44.05	8.30	1.75	0.50	0.27
S13	11.12	6.78	4.74	3.18	36.69	7.97	1.61	0.16	0.14
S14	15.29	10.96	6.31	3.90	37.27	9.77	1.58	1.35	0.79
S15	14.69	8.83	4.24	2.78	41.55	9.21	1.96	0.18	0.14
S16	16.91	7.64	7.05	4.73	43.77	11.76	1.53	1.12	0.75
S17	16.30	7.66	7.14	4.67	43.94	11.92	1.75	0.84	0.44
S18	17.08	8.52	4.34	2.69	38.10	9.00	1.92	0.17	0.15
S19	16.78	7.81	7.42	4.76	44.87	12.64	1.98	0.18	0.15
S20	17.06	7.59	7.22	4.75	43.91	12.00	1.65	1.04	0.76

Table S3. Information summary of tested samples

Sample No.	Coptidis Rhizoma (producing area)	Euodiae Fructus (producing area)
S1	CR7 (Shizhu, Chongqing City)	EF7 (Zhangshu, Jiangxi Prov.)
S2	CR1 (Lichuan, Hubei Prov.)	EF1 (Yiyang, Hunan Prov.)
S3	CR2 (Lichuan, Hubei Prov.)	EF2 (Yiyang, Hunan Prov.)
S4	CR3 (Lichuan, Hubei Prov.)	EF3 (Yiyang, Hunan Prov.)
S5	CR4 (Emeishan, Sichuan Prov.)	EF4 (Ji’an, Jiangxi Prov.)
S6	CR5 (Emeishan, Sichuan Prov.)	EF5 (Ji’an, Jiangxi Prov.)
S7	CR6 (Emeishan, Sichuan Prov.)	EF6 (Ji’an, Jiangxi Prov.)
S8	CR8 (Shizhu, Chongqing City)	EF8 (Zhangshu, Jiangxi Prov.)
S9	CR9 (Shizhu, Chongqing City)	EF9 (Zhangshu, Jiangxi Prov.)
S10	CR10 (Shizhu, Chongqing City)	EF10 (Zhangshu, Jiangxi Prov.)
S11	CR11 (Shizhu, Chongqing City)	EF11 (Zhangshu, Jiangxi Prov.)
S12	CR12 (Shizhu, Chongqing City)	EF12 (Zhangshu, Jiangxi Prov.)
S13	CR13 (Lichuan, Hubei Prov.)	EF13 (Yiyang, Hunan Prov.)
S14	CR14 (Lichuan, Hubei Prov.)	EF14 (Yiyang, Hunan Prov.)
S15	CR15 (Lichuan, Hubei Prov.)	EF15 (Ji’an, Jiangxi Prov.)
S16	CR16 (Emeishan, Sichuan Prov.)	EF16 (Ji’an, Jiangxi Prov.)
S17	CR17 (Emeishan, Sichuan Prov.)	EF17 (Zhangshu, Jiangxi Prov.)
S18	CR18 (Emeishan, Sichuan Prov.)	EF18 (Zhangshu, Jiangxi Prov.)
S19	CR19 (Shizhu, Chongqing City)	EF19 (Zhangshu, Jiangxi Prov.)
S20	CR20 (Shizhu, Chongqing City)	EF20 (Zhangshu, Jiangxi Prov.)
